# Error-Prone ZW Pairing and No Evidence for Meiotic Sex Chromosome Inactivation in the Chicken Germ Line

**DOI:** 10.1371/journal.pgen.1002560

**Published:** 2012-03-08

**Authors:** Silvana Guioli, Robin Lovell-Badge, James M. A. Turner

**Affiliations:** Division of Stem Cell Biology and Developmental Genetics, Medical Research Council, National Institute for Medical Research, London, United Kingdom; Washington State University, United States of America

## Abstract

In the male mouse the X and Y chromosomes pair and recombine within the small pseudoautosomal region. Genes located on the unsynapsed segments of the X and Y are transcriptionally silenced at pachytene by Meiotic Sex Chromosome Inactivation (MSCI). The degree to which MSCI is conserved in other vertebrates is currently unclear. In the female chicken the ZW bivalent is thought to undergo a transient phase of full synapsis at pachytene, starting from the homologous ends and spreading through the heterologous regions. It has been proposed that the repair of the ZW DNA double-strand breaks (DSBs) is postponed until diplotene and that the ZW bivalent is subject to MSCI, which is independent of its synaptic status. Here we present a distinct model of meiotic pairing and silencing of the ZW pair during chicken oogenesis. We show that, in most oocytes, DNA DSB foci on the ZW are resolved by the end of pachytene and that the ZW desynapses in broad synchrony with the autosomes. We unexpectedly find that ZW pairing is highly error prone, with many oocytes failing to engage in ZW synapsis and crossover formation. Oocytes with unsynapsed Z and W chromosomes nevertheless progress to the diplotene stage, suggesting that a checkpoint does not operate during pachytene in the chicken germ line. Using a combination of epigenetic profiling and RNA–FISH analysis, we find no evidence for MSCI, associated with neither the asynaptic ZW, as described in mammals, nor the synaptic ZW. The lack of conservation of MSCI in the chicken reopens the debate about the evolution of MSCI and its driving forces.

## Author Summary

In meiosis, the correct segregation of the homologous chromosomes is assured by the coordination of synapsis (a pairing mechanism) and repair of DNA double-strand breaks through homologous recombination. The sex chromosomes represent a challenge to the system, because they are largely comprised of non-homologous sequences. In mouse, the X and Y pair and recombine within a small region of homology. During pachytene, genes on the unsynapsed portions are repressed, a phenomenon called Meiotic Sex Chromosome Inactivation (MSCI). In chicken, the female is the heterogametic sex, i.e. has ZW sex chromosomes, while males are ZZ. In order to determine whether MSCI is conserved in this species, we analysed ZW synaptic behaviour and transcriptional activity. We found that ZW synapsis normally occurs through homologous and heterologous regions, and it is disassembled at diplotene (the stage following pachytene). A high number of oocytes fail to achieve ZW synapsis and recombination, but are nevertheless carried over to diplotene. We did not find evidence of MSCI. We favour a model whereby the sex chromosomes and the autosomes undergo transcriptional downregulation at the beginning of meiosis, which is maintained into diplotene. These data raise questions about checkpoint controls and have repercussions on theories of MSCI evolution.

## Introduction

Meiosis is the process in which maternal and paternal homologous chromosomes engage in close physical pairing, termed synapsis, and exchange genetic information by recombination through the programmed formation and repair of DNA double-strand breaks (DSBs) [1]. Autosomal homologs synapse and recombine along their entire length, but the sex chromosomes are often largely non-homologous, or, in some species, even lack a pairing partner [2] and therefore represent a challenge to a system based on homologous pairing and recombination. The strategy adopted by the sex chromosomes to assure correct segregation in the heterogametic sex varies greatly among different groups and this variability is present even within mammals [3], [4]. In mouse the X and Y are highly divergent in gene content and size, and synapsis and recombination is restricted to a distal limited region of homology, called the pseudoautosomal region (PAR) [5]. The X and Y chromosomes therefore remain largely unsynapsed throughout much of meiosis in normal males.

Genes located on the unsynapsed regions of the X and Y chromosomes are transcriptionally silenced at early pachytene by Meiotic Sex Chromosome Inactivation (MSCI) [6], [7], [8]. Several studies have provided evidence that MSCI is driven by the unsynapsed state of the XY pair [9], [10], [11]. MSCI is thought to represent a manifestation of a more general mechanism that silences any chromosome that fails to synapse with its homologue, termed Meiotic Silencing of Unsynapsed Chromatin (MSUC) [12]. The function of meiotic silencing is unclear, but it has been suggested to prevent ectopic recombination events between non-homologous sequences [6], to cull cells with synaptic errors [7], and/or to halt gene expression at sites of DNA damage [13].

Meiotic silencing was described originally in the fungus *Neurospora* [14] and later in members of the animal kingdom, including *C. elegans* [15], mice [9], [11], humans [16] and *D. melanogaster* [17], [18] (but see also [19]). Nevertheless, the effectors that mediate silencing in different model organisms vary considerably. For instance, meiotic silencing in mice is dependent upon BRCA1 [20], MDC1 [21] and phosphorylation of the histone variant H2AFX (forming γH2AFX) [22], while in *C. elegans* it requires components of the RNA-interference pathway [23], [24]. Furthermore, in mammals, meiotic silencing occurs at the transcriptional level [9], [11], while in *Neurospora* it occurs post-transcriptionally [14]. These mechanistic dissimilarities are consistent with a model in which meiotic silencing arose multiple times during evolution, perhaps serving distinct functions in different organisms. A key unresolved question is when during evolution the H2AFX-dependent form of silencing currently extant in mammals arose. A recent study found that MSCI operates in the male germ line of the marsupial *Monodelphis domestica*, and that the inactive marsupial XY bivalent is enriched for γH2AFX [25]. This finding places the appearance of the H2AFX-dependent silencing mechanism prior to the eutherian/metatherian radiation, 148 Mya. Clearly, analysis of other, more distantly related model organisms is now essential.

A good example of such an organism is the chicken. The avian and mammalian lineages diverged around 300 Mya. In contrast to mammals, females are the heterogametic sex in birds. Unlike the XY pair, which remains largely unsynapsed throughout the duration of pachytene, classical EM studies have described the pairing status of the chicken ZW pair as highly dynamic during pachytene [26], [27]. The Z and W are thought to associate at their homologous ends at early pachytene and to undergo a subsequent stage of complete synapsis involving their non-homologous regions. ZW desynapsis was reported to occur during late pachytene, leaving the Z and W associated only at their homologous extremities.

Moreover, a recent study proposed that DNA DSB markers on the ZW pair disappear at synapsis, but reappear later during desynapsis and are carried over to the diplotene stage [28]. Importantly this study reported that MSCI occurs during chicken oogenesis. A model was suggested in which the W chromosome enters meiosis in an already inactive state, and that inactivation subsequently spreads *in trans* to the Z chromosome via meiotic pairing [28].

Here, we address both the dynamics of ZW synapsis and the transcriptional state of the ZW pair during chicken meiotic progression, using existing and novel synapsis and recombination markers as well as gene-specific RNA FISH. In contrast to previous reports, we find that ZW DNA DSB foci disappear at synapsis and that ZW desynapsis normally occurs at diplotene. Unexpectedly we found that the process of synapsis is highly error prone. Furthermore, we find no evidence to support the existence of a pachytene-specific wave of ZW chromosome silencing. Our findings have important implications for our understanding of the evolution of meiotic silencing.

## Results

### A population of chicken oocytes never achieve ZW synapsis

**Figure 1:**
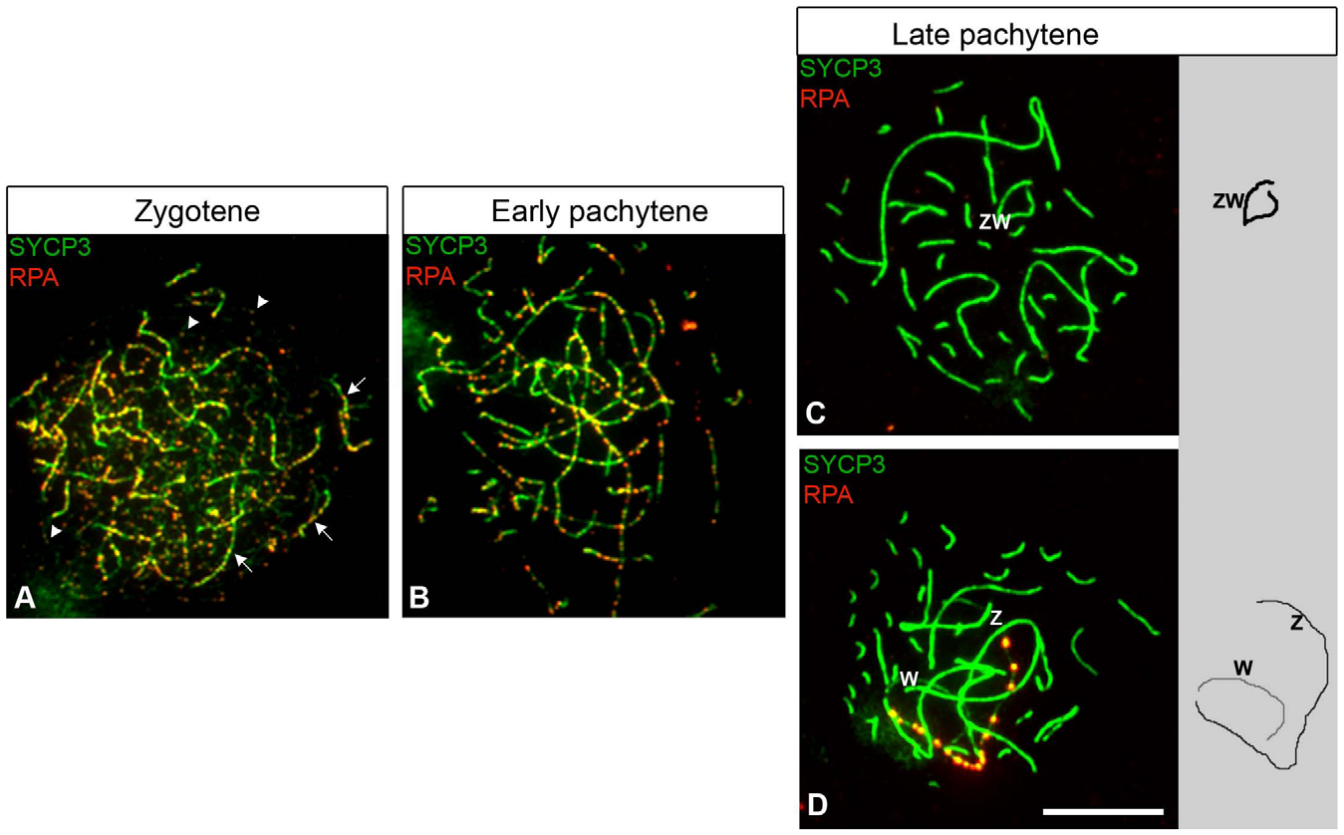
RPA localization in chicken oocytes. Oocytes stained for RPA (red) and SYCP1/SYCP3 (green). RPA and SYCP1 co-staining was performed first (Figure S1). After imaging, the slides were then stained for SYCP3 and new pictures were taken. (A) Zygotene, RPA foci are on synapsed and unsynapsed chromosomes (indicated with arrows and arrowheads, respectively). (B–D) Pachytene; RPA foci on autosomal synaptic axes are still abundant at early pachytene (B), but rapidly decrease and then disappear by late pachytene (C–D). Pachytene cells were classified as late if they contained fewer than five foci on synaptic autosomes. (C) Late pachytene oocyte containing a synapsed ZW bivalent RPA negative. (D) Late pachytene oocyte containing an unsynapsed Z and W carrying chains of RPA foci on the Z. Z and W were identified by hybridisation to Z and W chromosome paints (data not shown). Drawings of ZW synaptic configurations are shown to the right of panel C and D. Scale bar = 10 µm.

During oogenesis, germ cells progress through meiosis in a broadly synchronised wave. In order to follow the synaptic behaviour of the ZW pair, we isolated ovaries at embryonic day (E) 19 and at 0, 1, 3 and 6 days post-hatching (dph), the time period during which, in our stock, germ cells progress from leptotene to diplotene. To assess synapsis at each of these stages, we immunostained oocytes with antibodies to the axial element marker SYCP3 [29] and the recombination protein RPA (Figure 1) [30], [31], [32]. In male (XY) mice, RPA appears on axes prior to synapsis, accumulates on newly-synapsed autosomal bivalents at early pachytene, and subsequently disappears from these sites during the early to late pachytene transition, concomitant with the resolution of meiotic DNA DSBs [31], [32]. In contrast, RPA staining persists on the asynapsed X chromosome untill late pachytene [31]. We therefore envisaged that RPA would be a useful marker to follow the synaptic behaviour of the Z chromosome during chicken oogenesis.

The time course of RPA localization on autosomes in chicken oocytes was similar to that observed in mouse spermatocytes: RPA foci were abundant until early pachytene (Figure 1A, 1B), but then reduced in number with few or no foci evident by late pachytene (Figure 1C). Using this RPA staining criterion, we then focused on the late pachytene oocyte population, expecting to find evidence of ZW desynapsis as previously suggested [26].

**Figure 2:**
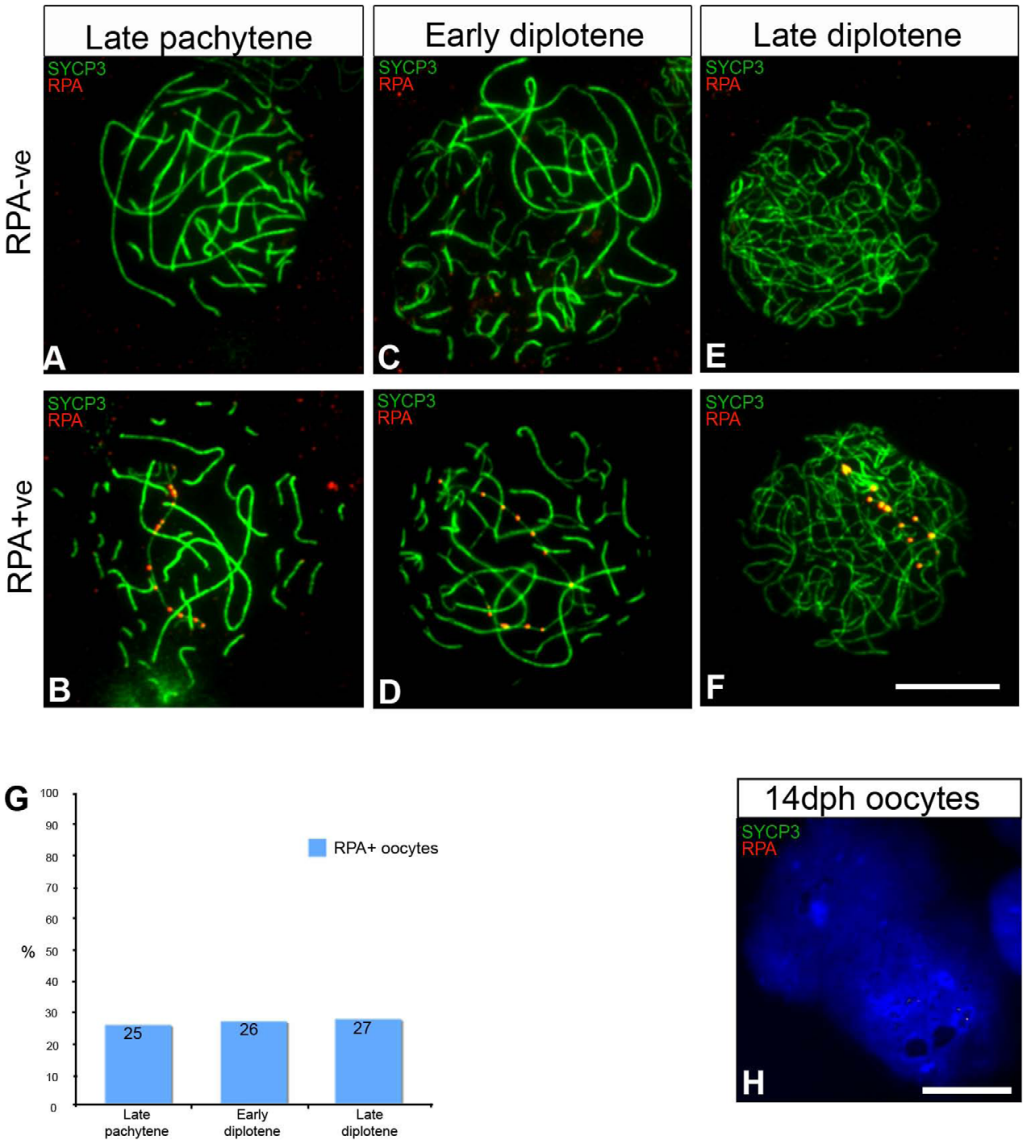
Oocytes with unresolved DNA DSBs on the Z chromosome progress to diplotene. (A–F) 6 dph oocytes stained for RPA (red) and SYCP3 (green). The diplotene cells were classified as early when desynapsis was partial and late when desynapsis was complete. (A–B) Late pachytene; (C–D) early diplotene; (E–F) late diplotene. (A, C, E) Cells RPA negative (RPA^−ve^), and (B, D, F) cells carrying a chain of RPA foci (RPA^+ve^). (G) Graph showing the percentage of RPA^+ve^ cells at each stage. Total number of cells analysed: late pachytene, 141; early diplotene, 223; late diplotene, 169. ZW chromosome painting performed after antibody staining showed that the Z chromosome accounted for 85% of the RPA^+ve^ cells identified at pachytene and 74% at early diplotene, making the percentage of late pachytene ZW^RPA+^ = 21.7% and the percentage of early diplotene ZW^RPA+^ = 19.6%. The percentage of ZW^RPA+^ pairs at late diplotene was not calculated as in many of these cells it was not possible to unambiguosly identify the entire Z axis. The quantification was done on ovaries from the chicken line ISA Brown. (H) 14 dph oocytes stained for RPA (red) and SYCP3 (green). 90% of the cells are negative for SYCP3; no RPA was identified in these cells (number of cells analysed: 60). Scale bar = 10 µm.

We identified late pachytene oocytes with unsynapsed sex chromosome pairs, each exhibiting chains of RPA foci along the axial length of the Z (Figure 1D). These were confirmed as unsynapsed using RPA/SYCP1 combined staining (Figure S1). However, two observations were inconsistent with this sex chromosome configuration arising through desynapsis. Firstly, the percentage of cells containing such “separated ZW pairs”, hereafter termed ZW^RPA+^ pairs, was low, representing less than a quarter of the total late pachytene oocytes examined (see legend to Figure 2). Secondly, the RPA foci on ZW^RPA+^ pairs were unusually strong in intensity compared to the foci observed on the Z and W axes at early pachytene (Figure 1B, 1D). As accumulation of DNA DSB proteins on chromosome axes is a characteristic sign of synaptic failure [33], [34], we considered the hypothesis that ZW^RPA+^ pairs had never achieved synapsis during pachytene and that this resulted in the retention and accumulation of DNA DSB-associated markers. We also noted that RAD51 persisted on the ZW^RPA+^ pairs (Figure S2).

To test our ‘ZW asynapsis’ model we examined the proportion of oocytes exhibiting ZW^RPA+^ pairs as meiosis progressed from late pachytene to diplotene (Figure 2A–2F). We subdivided diplotene oocytes into early and late substages according to the degree of autosomal desynapsis revealed by SYCP3 and scored the percentage of oocytes with persistent RPA foci chains in all three populations (Figure 2G, see legend for description). We reasoned that if ZW^RPA+^ pairs resulted from late pachytene desynapsis, the proportion of oocytes with RPA foci would drastically increase by diplotene. In contrast, we found that the proportion was unchanged over this period, remaining between 25 and 27% (Figure 2G). This observation is consistent with a model in which ZW^RPA+^ pairs represent asynapsed bivalents. This also shows that oocytes with asynapsed ZW pairs progress through pachytene and diplotene. RPA foci eventually disappeared by 14 dph, when oocytes had lost SYCP3 staining (Figure 2H, see legend for details).

**Figure 3:**
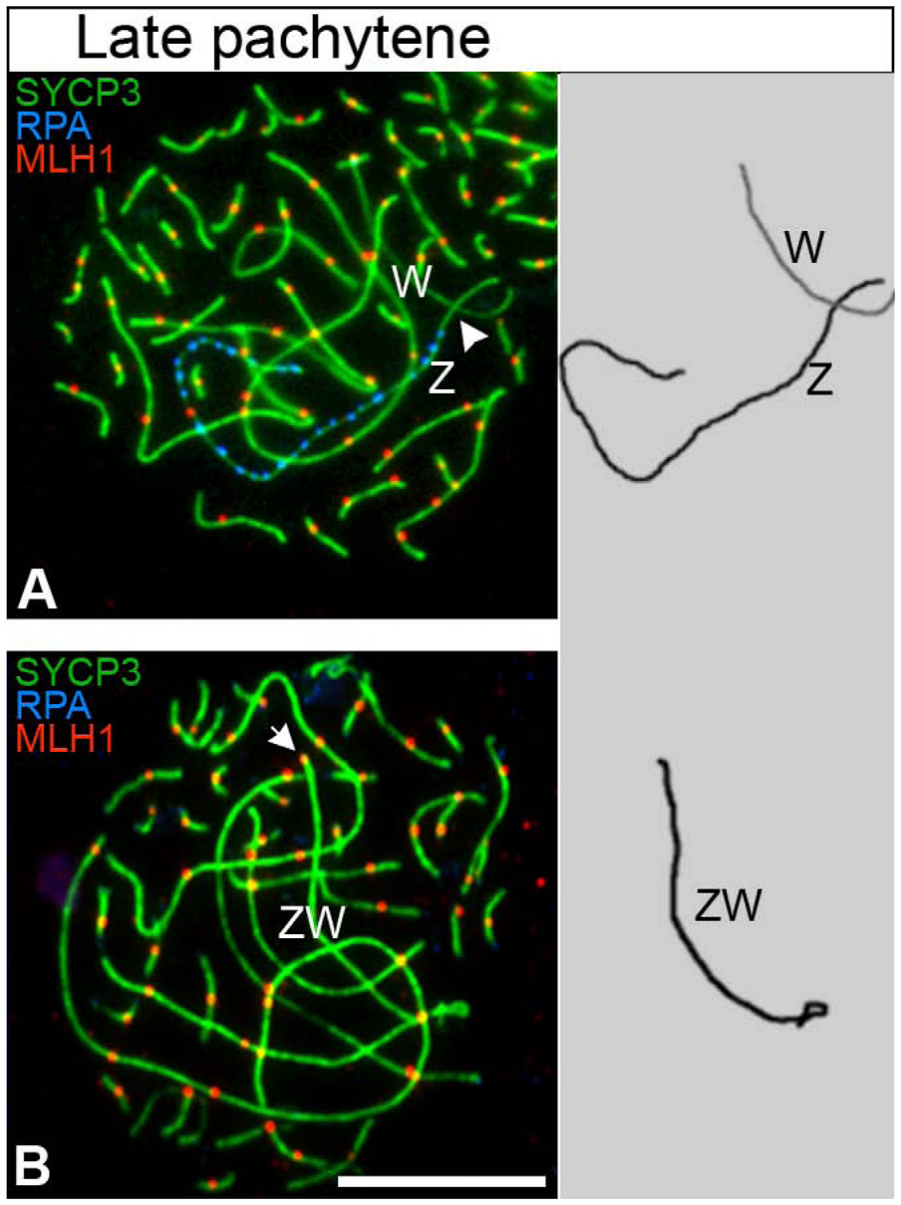
The late pachytene ZW^RPA+^ pair has no MLH1 focus. Oocytes triple stained for SYCP3 (green), RPA (blue) and MLH1 (red). (A) The ZW^RPA+^ pair carrying a chain of RPA foci on the Z never has a MLH1 focus (white arrowhead indicates the expected location of the focus); total number of oocytes analysed: 62. (B) The fully synapsed ZW bivalent always carries a MLH1 focus at one end (white arrow); Total number of oocytes analysed: 138. Drawings of the ZW pair to the right of ech panel. ZW pairs were identified using ZW chromosome paints (data not shown). Scale bar = 10 µm.

To further test if ZW^RPA+^ pairs observed at late pachytene were asynaptic rather than desynaptic, we combined immunostaining for SYCP3/RPA with that of MLH1, a component of the obligatory crossover that forms at the PAR [35], [36]. Crossovers occur after synapsis and recombination between homologous sequences. If late pachytene ZW^RPA+^ pairs had engaged in synapsis, they should thereafter remain tethered at their distal PAR by a crossover. We found that late pachytene ZW^RPA+^ pairs were never marked by MLH1 (Figure 3A), while stage-matched, fully synapsed ZW bivalents always exhibited a PAR-associated MLH1 focus (Figure 3B). Moreover we noticed that in some ZW^RPA+^ pairs Z and W axes were not in close proximity to each other. Thus, ZW^RPA+^ pairs result from asynapsis.

**Figure 4:**
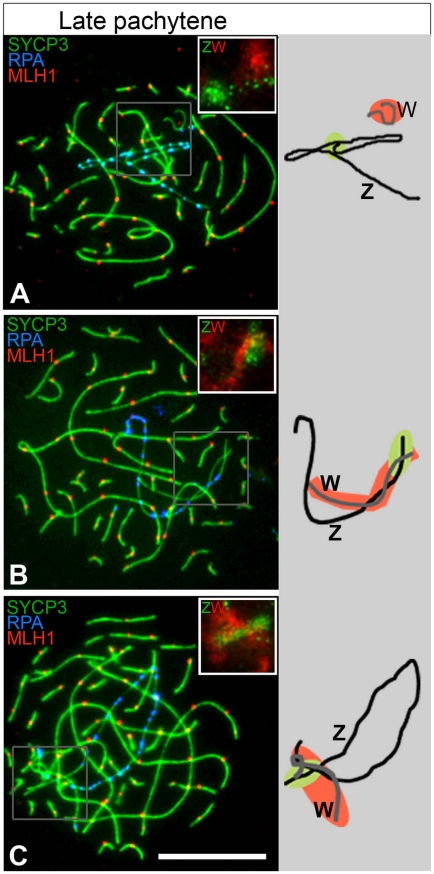
Many ZW^RPA+^ pairs have PAR-PAR misalignment. Late pachytene oocytes triple-stained for SYCP3 (green), RPA (blue), MLH1 (red) and subsequently hybridised to Z and W chromosome paints (top right insets: Z, green cloud; W, red cloud). Total number of ZW^RPA+^ oocytes analysed: 134. (A) Z and W axes are clearly separated; (B) only the q-end of the Z axis is close to the W; (C) both ends of the Z are close to the W (see text for percentages of each class). The ZW bivalents are also schematised to the right (black line = Z; grey line = W; green = Z paint; red = W paint). Scale bar = 10 µm.

The Z and W chromosome paints used in our experiments hybridize to large heterochromatic regions of the sex chromosomes; in the case of the Z chromosome this region mainly resides at the tip of the long (q) arm, i.e. at the opposite end to the PAR, while in the case of the W chromosome it dominates much of the chromosome length. The Z paint is therefore ideal to distinguish the two ends of the Z in pachytene and early diplotene cells and to allow us to investigate spatial associations between the Z and W chromosomes in more detail. At late pachytene we observed complete spatial separation of the Z and W axes in 11% of ZW^RPA+^ pairs (Figure 4A). In a further 55%, the W chromosome was in close proximity to one end of the Z chromosome. However, chromosome painting revealed that in 92% of these oocytes this was actually the heterochromatic q end, rather than the expected PAR end of the Z chromosome (Figure 4B). In the remaining bivalents an assessment of PAR-PAR interactions was impossible because both ends of the Z chromosome were in close proximity to the W chromosome (Figure 4C). Together, these findings show that at least 60% of late pachytene ZW^RPA+^ pairs display complete failure in PAR-PAR association, confirming that those ZW^RPA+^ pairs are asynaptic.

### ZW synapsis is not transient during pachytene

**Figure 5:**
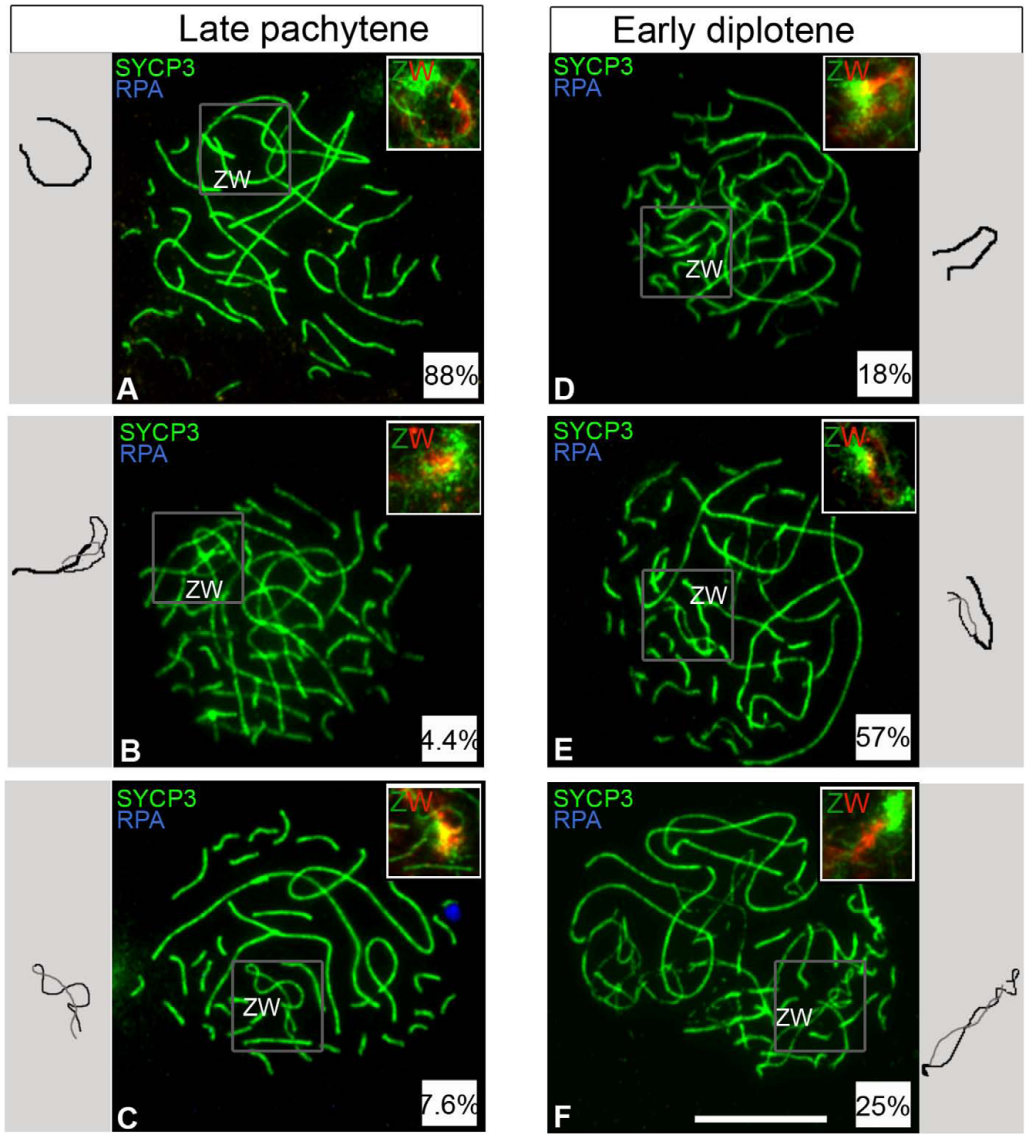
ZW bivalent desynapsis occurs in synchrony with autosomal desynapsis. Oocytes double-stained for SYCP3 (green) and RPA (blue) and subsequently hybridised to Z and W chromosome paints (green and red clouds respectively in top right insets); only the late pachytene and early diplotene cells negative for RPA were considered. (A–C) Late pachytene cells (total number analysed: 134); (A) ZW is fully synapsed; (B) ZW is partially desynapsed; (C) Z and W are fully desynapsed. (D–F) Early diplotene cells (total number analysed: 54); (D) ZW is fully synapsed; (E) ZW is partially desynapsed; (F) Z and W are fully desynapsed. Relative percentages are indicated in the insets at bottom right corner of each panel. To the side of each panel is a schematic of the ZW pair (Thick black line, synapsed ZW; black and grey thin lines, desynapsed Z W, respectively). In most pachytene cells the ZW pair is synapsed, in most early diplotene cells ZW is partially desynapsed. Scale bar = 10 µm.

Our results so far established that one fifth of oocytes never achieve ZW synapsis during pachytene. We next wished to determine the timing of ZW desynapsis in the remaining late pachytene oocytes in which ZW synapsis and DNA DSB repair had occurred normally. We identified these oocytes by their negativity for Z chromosome-associated RPA foci. Analysis of 6 dph ovaries revealed complete ZW synapsis in 88% of late pachytene oocytes (Figure 5A). A further 4.4% exhibited partially desynapsed ZW bivalents (Figure 5B) and the remaining 7.6% exhibited end-to-end ZW associations, i.e. complete desynapsis (Figure 5C).

The existence of such a small percentage of desynapsed ZW bivalents at late pachytene suggested two hypotheses: either most ZW bivalents do not undergo premature desynapsis at late pachytene, or premature desynapsis occurs in most oocytes, as previously reported [26], but it is restricted to a narrow time window at the very end of late pachytene. In the latter scenario, the vast majority of ZW bivalents would be expected to exhibit end-to-end associations during subsequent early diplotene. Contrary to this model, only 25% of early diplotene oocytes exhibited end-to-end associations while 18% and 57% showed complete synapsis and partial desynapsis, respectively (Figure 5D–5F). We conclude that most ZW bivalents do not initiate premature desynapsis during pachytene.

### A lack of inactivating marks associated with the meiotic Z chromosome

**Figure 6:**
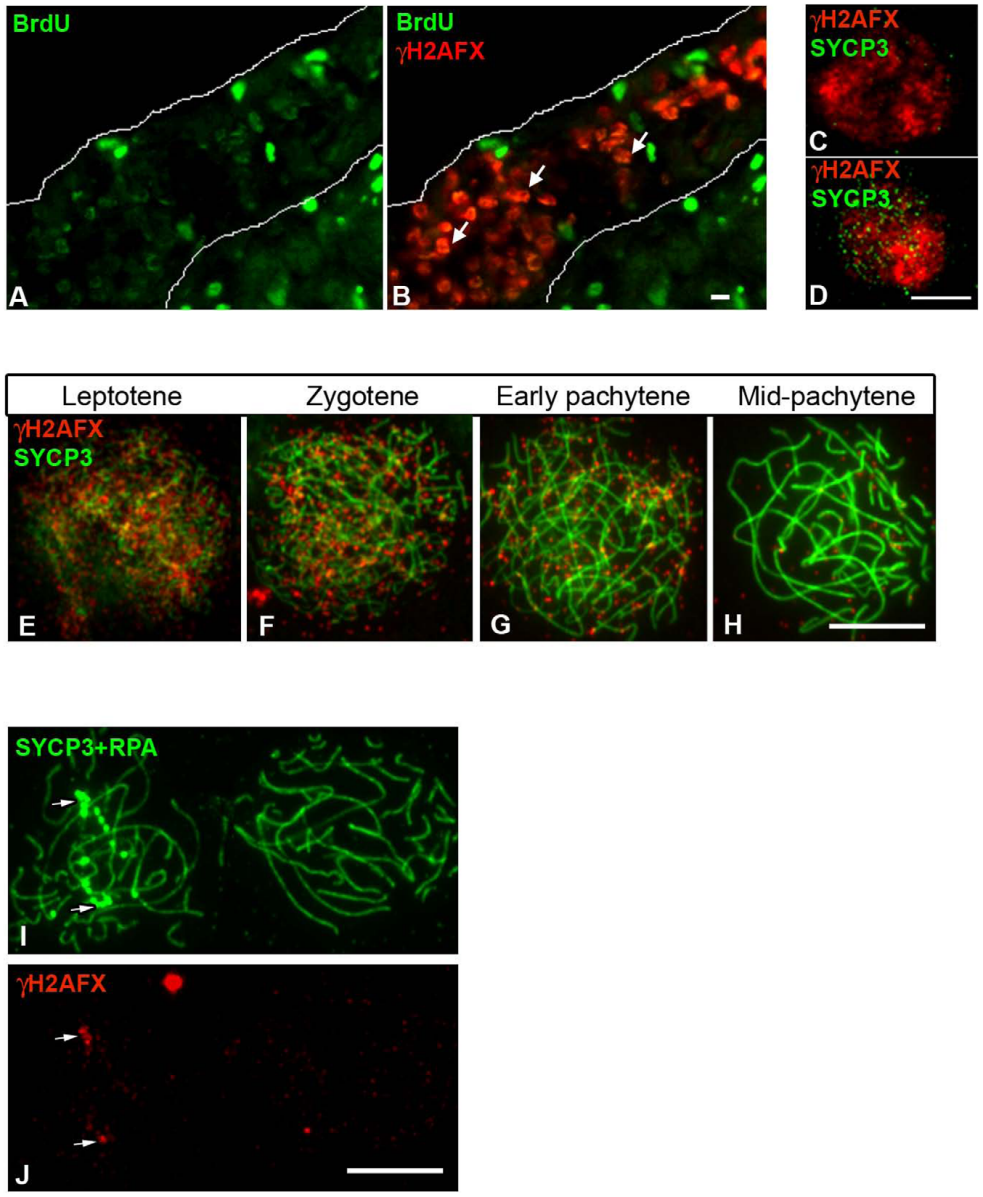
Chick oocytes undergo a wave of γH2AFX coincident with the beginning of meiosis. (A–B) Ovarian section from E14 embryos injected with BrdU two hours before dissection and double-stained for BrdU (green) and γH2AFX (red). Many germ cells are positive for both markers (e.g. white arrows). (C–D) Oocyte nuclei from E14 ovarian spreads double-stained for γH2AFX (red) and SYCP3 (green). SYCP3 is only present in some of the γH2AFX positive cells. (E–H) γH2AFX timecourse on ovarian spreads. The cells were double-stained with SYCP3 for staging. γH2AFX is downregulated by mid-pachytene. (I–J), Pachytene cells triple-stained for SYCP3 (green), RPA (green) and γH2AFX (red): γH2AFX is only present in the cell containing asynaptic RPA+ve chromosomes (left nucleus), where it colocalises with some RPA foci (white arrows). Scale bar = 10 µm.

Next, we looked at the chicken ZW bivalent for the presence of inactivating chromatin marks found previously to be associated with MSCI in the mouse. One such modification is H2AFX phosphorylation [22], [37]. We found that in chicken, like in mouse, a wave of H2AFX γ-phosphorylation appears at meiotic S-phase, being evident in BrdU positive germ cells at E14 (Figure 6A–6B). γH2AFX was also observed throughout the nucleus in developmentally advanced cells, identified as leptotene by their positivity for SYCP3 (Figure 6C–6D). As in mice, this DNA DSB-related staining was maintained through zygotene, before vanishing by mid pachytene (Figure 6E–6H). Notably, in contrast to the mouse XY bivalent, chromatin-wide γH2AFX staining was not found on the asynaptic ZW^RPA+^ pairs at pachytene. γH2AFX was only found in association with the axis of these ZW^RPA+^ pairs, where it remained at unresolved DNA DSBs, marked by strong RPA foci (Figure 6I–6J). Thus, a sex chromatin-wide wave of H2AFX γ-phosphorylation does not occur during chicken oogenesis. We also noted that another protein implicated in MSCI, TopBP1 [38], was restricted to foci on ZW^RPA+^ pairs, and was not evident along the full length of the axis or in the chromatin, in contrast to the situation in the mouse (Figure S3).

**Figure 7:**
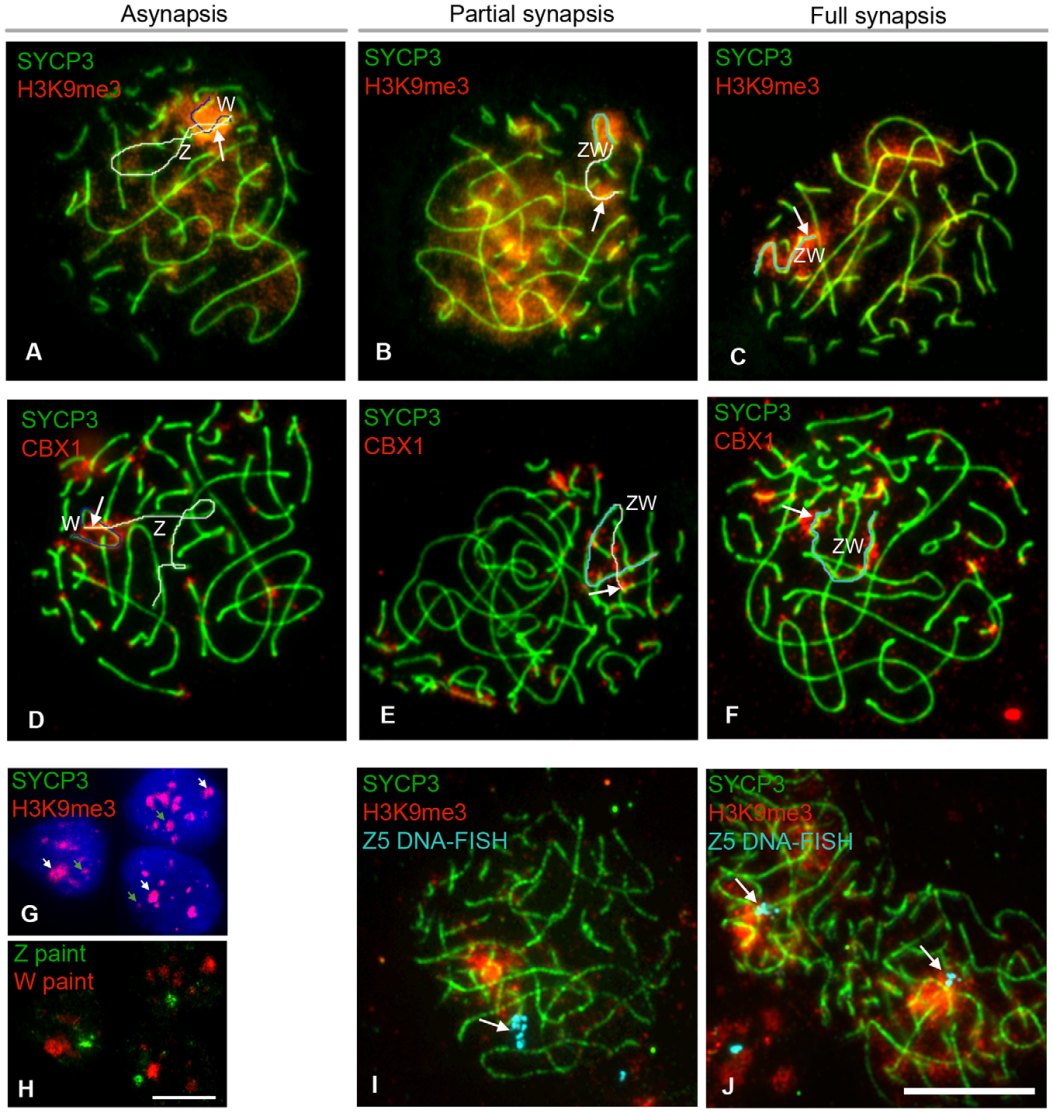
Epigenetic status of Z and W chromosomes at pachytene. (A–F) Oocyte nuclei double stained for SYCP3 (green) and H3K9me3 (A–C), or CBX1 (D–F) (red). Z and W have been identified by chromosome paint (data not shown). Asynapsed Z and W have been pseudo-coloured white and blue, respectively; synapsed ZW has been pseudo-coloured turquoise. Both markers stain the W chromatin, and the heterochromatic q end of the Z (indicated with a white arrow). (G–H) Ovarian somatic cells stained for H3K9me3 (red) (G), and subsequently hybridised to Z (green) and W (red) chromosome paints (H). Z and W chromatin are always H3K9me3 positive (green and, respectively, white arrows in G); total number of cells analysed: 67. (I–J) Oocyte nuclei double-stained for SYCP3 (green) and H3K9me3 (red), and subsequently processed for DNA-FISH using BAC CH261-125H16 (Z5) as a probe. This BAC contains ∼200KB of Z DNA, including the gene Dmrt1, located at 27 Mb on the chicken Z physical map (www.ensemble.org). The signal (turquoise) is indicated by a white arrow. The probe was found to localise outside the H3K9me3 domain (I) or to the domain border (J) in 89% of the synapsed ZW pairs analysed; total number of cells analysed: 40. Scale bar = 10 µm.

We then studied the localization of other MSCI-associated chromatin marks: CBX1 [39] and H3K9me3 [40], [41]. We found that both marks were enriched at the pericentromeric chromatin of all chromosome bivalents, as well as being localised to the Z and W chromosomes (Figure 7A–7F). H3K9me3 was not specific to the sex chromatin as several domains were observed localising to different chromosomes (Figure 7A–7C). Closer examination of late pachytene cells revealed that both marks were restricted to the heterochromatic block on the q-arm of the Z chromosome. The W chromatin was more extensively covered in both marks, with CBX1 being enriched in discrete domains, again consistent with these marks being components of constitutive heterochromatin. Importantly, no significant difference in this localisation pattern was observed between ZW bivalents that were synapsed or asynapsed.

These data suggested that the association of H3K9me3 and CBX1 with the Z and W chromosomes may be unrelated to their synaptic status or to any potential meiotic silencing phenomenon, and may instead merely reflect the presence of blocks of constitutive heterochromatin. We therefore analysed their patterns in somatic ovarian supporting cells. Although CBX1 was not detected, the whole W chromosome and the Z chromosome heterochromatic q block, identified by chromosome painting, were H3K9me3 positive in these cells, consistent with this hypothesis (Figure 7G–7H). Furthermore, DNA FISH on pachytene oocytes revealed that DNA from the euchromatic portion of the Z chromosome was located outside or on the border of the H3K9me3 domain (Figure 7I–7J).

### Expression of Z-linked genes persists into pachytene and is not biased by the synaptic status of the ZW bivalent

**Figure 8:**
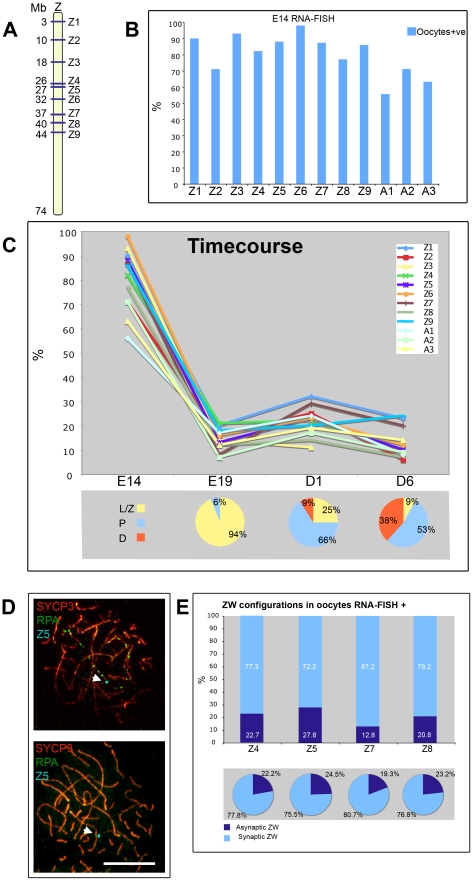
A global downregulation of transcription occurs at leptotene-zygotene and is maintained into pachytene and early diplotene. (A) Schematic of the Z chromosome showing the location of the Z probes used in the analysis. (B) RNA-FISH analysis using Z (Z1–Z9) and autosomal (A1–A3) probes on oocytes from E14 (see Materials and Methods for probe identity). The oocytes were identified by subsequent staining for γH2AFX. The bars represent the percentage of expressing oocytes. (C) (Top) Timecourse results using the probes screened at E14and subsequently analysed at E19, 1 dph (D1), 6 dph (D6); the oocytes were identified by staining for SYCP3. The Y axis indicates the percentage of expressing oocytes. (C) (Bottom) Charts representing the percentage of leptotene/zygotene (L/Z), pachytene (P) and diplotene (D) cells in the ovaries analysed at E19, D1 and D6; the counting was carried out on spreads generated from the cell dispersions used for RNA-FISH, after staining for SYCP3 and RPA. (D) Oocyte nuclei from ovarian spreads subject to RNA-FISH using the probe Z5 (cyan) and stained for SYCP3 (red) and RPA (green); (Top) Positive oocyte containing ZW^RPA+^; (Bottom) Positive oocyte containing a fully synapsed ZW. The positive signal (cyan) is also indicated by a white arrow. Scale bar = 10 µm. (E) Plots summarising the results from the RNA-FISH on spreads using probes Z4, Z5, Z7, Z8. (Top) Relative percentages of synaptic and asynaptic ZW within the positive late pachytene oocytes. (Bottom) Chart representing the relative percentages of synaptic and asynaptic ZW within the total population of late pachytene oocytes. The activity of the pachytene ZW pair is not biased by its synaptic status.

Previous work has shown that in mouse spermatocytes global transcription levels drop considerably during leptotene and zygotene [11]. At the onset of pachytene, the autosomes recover their transcriptional activity, while the X and Y are subject to MSCI [42]. Equivalent analyses in chicken oocytes are lacking, so we decided to follow autosome and sex chromosome-specific activity during chicken oogenesis. We used RNA FISH for this purpose, to allow us to visualise nascent transcripts on a cell-by-cell basis. We initially screened for genes expressed in E14 ovaries, when most cells are in pre-leptotene, i.e. prior to the onset of meiotic prophase. We found nine Z-linked (Figure 8A) and three autosomal BACs containing-genes expressed at this stage, and in each case RNA signals were observed in the majority of cells studied (Figure 8B). At E19, when most cells are in leptotene and zygotene, the transcriptional activity of all genes was drastically down-regulated, being expressed in a lower percentage of oocytes than at E14. Subsequently at 1 dph and at 6 dph, when cells progress through pachytene and diplotene, the proportion of cells positive for each probe signal remained relatively low. Of note, the range of autosomal activity was 17–24% at 1 dph and 8–14% at 6 dph, and the activity of genes on the Z chromosome followed a similar trend, the range being 11–32% at 1 dph and 6–25% at 6 dph (Figure 8C). These data suggest that in chicken, as in mouse, there is a global downregulation of transcription in early prophase [42], but in chicken this seems to be maintained through pachytene and early diplotene.

Importantly, the finding that Z-gene expression can be observed during pachytene argues against the existence of a wave of sex chromosome silencing analogous to that seen in mammals. However, since silencing might be expected to occur only on particular sex chromosome configurations, we could have missed subtle changes in Z chromosome expression using this approach. We therefore combined RNA-FISH on 1 dph oocytes with SCP3/RPA immunostaining in order to distinguish cells carrying the ZW^RPA+^ pairs. We found that both ZW^RPA+^ and ZW^RPA−^ cells were transcriptionally active for all the Z probes tested. Four of the FISH experiments (probes Z4,5,7,8) were performed on surface spreads in order to look directly for a correlation between the presence or absence of a Z-gene RNA FISH signal and the synaptic status of the Z chromosome (Figure 8D). The percentage of cells with synaptic and asynaptic ZW pairs expressing each of the four genes correlated well with the percentage of synaptic and asynaptic ZW within the populations (Figure 8E). We also observed a similar frequency of expression for both ZW^RPA+^ and ZW^RPA−^ cells for a further five Z-linked RNA FISH probes (probes Z1,2,3,6,9; Table S1).

Thus, the activity of each of the Z-genes studied are independent of the synaptic status of the Z chromosome. These data further argue against the existence of a pachytene-specific wave of Z chromosome silencing during chicken oogenesis.

## Discussion

### The dynamics of synapsis and DSB repair of the chicken ZW pair: a new model

In this study, we have investigated the synaptic behaviour and transcriptional status of the ZW bivalent during chicken meiosis, as a first step towards understanding the conservation and evolution of meiotic silencing in the animal kingdom.

**Figure 9:**
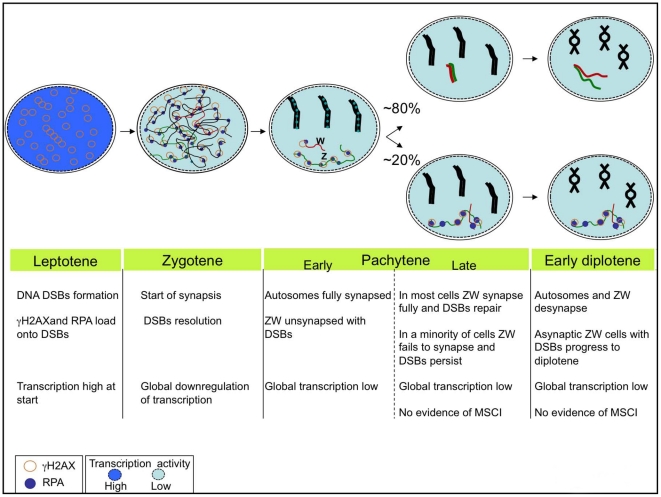
The chick sex chromosome meiotic synaptic behaviour: a new model. Schematic representing the dynamics of synapsis and DSB repair of the ZW pair during early meiosis. Autosome axes in black; Z in green; W in red. RPA foci are shown as blue dots, γH2AX as circles. In about 80% of the oocytes that reach pachytene ZW undergoes synapsis and DSB repair by mid-late pachytene and desynapses at diplotene. In 20% of oocytes ZW remains asynapsed and maintains unrepaired DSBs, nevertheless the cells go to diplotene. Different shades of blu represent different levels of transcriptional activity (dark blue: high, light blue: low). The oocytes undergo a global downregulation of transcriptional activity at leptotene/zygotene. At pachytene, no Z specific wave of silencing is evident in any of its configurations. Autosomes and Z chromosomes behave alike; they maintain a low level of transcription at least into early diplotene. See Discussion for more details.

Our findings lead us to a new model for the kinetics of ZW pairing in the chicken that challenges the currently accepted view. We propose that in chicken oocytes DSBs on the sex chromosomes are normally resolved by the end of pachytene. The repair correlates with the formation of a heterologous synapsis between Z and W, which is generally maintained throughout pachytene and is disassembled at the beginning of diplotene. In one fifth of oocytes, the sex chromosomes fail to achieve synapsis and DNA DSB repair. Oocytes carrying these asynapsed bivalents nevertheless proceed to the diplotene stage (Figure 9).

The fundamental finding that led us to this model was the identification of two types of “separated” ZW pairs at late pachytene: one replete with DSB foci on the Z axis (ZW^RPA+^) and one without DSB foci. Our data demonstrate that the ZW^RPA+^ pairs are asynaptic, unveiling an intrinsic error-prone nature of the system. The proportion of oocytes that fail to achieve ZW synapsis and DSB repair is unexpectedly high when compared with that in male mice, in which asynapsis of the heteromorphic XY pair occurs at between four and nine percent [43]. The high level of ZW univalence may result in part from a generally elevated level of chromosome pairing defects occurring in the female germ line [44]. However, the frequency of ZW asynapsis that we observed far exceeds that noted for the autosomes (around 5%), indicating that ZW synapsis in the female germ line is highly error prone, with many oocytes susceptible to ZW pairing disturbances and consequent failure to establish a crossover at the PAR.

In chicken, Z and W synapsis normally proceeds from the homologous ends and spreads through the heterologous regions [26]. Interestingly, most ZW^RPA+^ oocytes exhibiting misalignment of their PARs displayed close apposition of their heterochromatic regions. Heterochromatin is rich in repeat sequences; we therefore favour a model in which microhomology between Z and W repeats acts as an antagonising force to PAR-PAR pairing. The ZW^RPA+^ pair identified in this work is likely to correspond to the late pachytene “separated” ZW pair carrying DSB foci, interpreted recently as a desynaptic species [28]. The demonstration that ZW^RPA+^ is an aberrant asynaptic pair clarifies that in chick the DSB foci permanently disappear at synapsis. This is in agreement with work in mammals showing that non-homologous synapsis results in permanent disappearance of DNA DSB proteins [33].

The nature of the ZW^RPA+^ pair also raises issues regarding the conservation of a pachytene quality surveilance mechanism in vertebrates. It is known that in yeast, recombination and synapsis defects are monitored by a pachytene checkpoint [45]. Although it is possible that a pachytene checkpoint is operating in mammals [46], some observations do not sit comfortably with this concept [42]. Recently it has been proposed that MSCI failure as well as meiotic silencing of genes on unsynapsed chromosomes could cause pachytene arrest [42], [47]. Our findings in chicken show that oocytes harbouring unsynapsed ZW bivalents progress through pachytene despite the Z chromosome being replete with unresolved DNA DSBs. Either no checkpoint operates during the pachytene stage of chicken oogenesis to monitor asynapsis/unresolved DNA DSBs, or a checkpoint does operate, but the number of unresolved DNA DSBs on the Z is not sufficiently high to trigger it [48]. Alternatively, the unresolved DNA DSBs on unsynapsed Z chromosomes may trigger a checkpoint later, at the diplotene/metaphase I transition, analogous to the timing of operation of the G2/M checkpoint in mitosis. Our findings that RPA foci disappear by 14 dph is consistent with the possibility that oocytes carrying ZW^RPA+^ pairs are eliminated, but could also reflect repair of these DSBs, presumably through sister chromatid exchange.

In male mice, unpaired sex chromosomes trigger a spindle checkpoint at metaphase I that senses a lack of spindle tension resulting from defective crossover formation [42]. In females, in contrast, oocytes with a limited number of unsynapsed bivalents can pass through both meiotic divisions. This is thought to be because the spindle checkpoint is less efficient in females, and this in turn may contribute to the high levels of aneuploidy resulting from maternal meiotic errors [49]. A recent study has implicated a Y-linked gene, *Zfy2*, as an important factor that determines the male/female differences in the spindle checkpoint efficiency [50]. If asynaptic ZW pairs persist to the first meiotic division, it will be of great interest to determine whether they give rise to elevated sex chromosome aneuploidy in oocytes and embryos.

It was previously reported that ZW bivalents separate by late pachytene, before the dissociation of autosomal axes which marks the beginning of diplotene [26]. Our data indicate that this notion should be revised. Although it is true that desynaptic ZW bivalents were observed in late pachytene cells, the number of this type of cell was quite small. In fact they accounted only for a maximum of 12% in 6 dph ovaries. The quantitative timecourse following the progression of ZW synapsis into diplotene clearly showed that only a small percentage of diplotene oocytes contained fully desynaptic ZW pairs, while the bulk contained ZW pairs in the process of desynapsis or still fully synapsed. We therefore propose that the sex chromosomes normally desynapse in broad synchrony with the autosomes.

### Meiotic sex chromosome inactivation

As mammalian MSCI is thought to be a manifestation of MSUC [7], a more general mechanism of silencing affecting any unsynapsed pachytene chromatin, the idea that MSCI/MSUC may be conserved and preceded the evolution of mammalian X and Y is appealing. The finding in chicken that pachytene oocytes carrying asynapsed ZW bivalents progress through to diplotene suggests two alternative hypotheses in relation to the evolution of MSCI/MSUC: the phenomenon is conserved in chicken, but the silencing of Z and W genes is not critical for survival at pachytene and early diplotene, or MSCI/MSUC is not conserved.

Our analysis of markers of MSCI and of gene expression by RNA FISH find no evidence that MSCI/MSUC occurs in the chicken germ line. Although we cannot exclude the possibility that silencing does occur at other sex-linked loci, in mice and in marsupials MSCI is a pan-chromosomal event affecting all coding genes [25], [51], [52]; furthermore, the sex chromatin-wide H2AFX phosphorylation and H3K9 trimethylation that occurs at the onset of silencing in mammals [11], [53] was not seen on the ZW bivalent. Therefore, if MSCI/MSUC does occur in the chicken, it must proceed in a mechanistically distinct manner. In conclusion our data do not support the existence of a pachytene wave of transcriptional silencing either associated with the asynaptic ZW, like in mammalian MSCI [7], or to the synaptic/postsynaptic ZW, as recently proposed [28]. Instead they are consistent with a model in which oocytes undergo a global downregulation of transcription at the beginning of meiosis that is maintained through pachytene and at least early stages of diplotene (Figure 9).

In male mice, silencing of the X chromosome is tolerated by the evolution of autosomal ‘backups’ retroposed functional copies of X-linked genes that are expressed specifically during pachytene [54]. The absence of meiotic silencing in the chicken oocyte would allow continued expression of oogenesis-critical Z-linked genes and thereby progression through pachytene. This may explain why autosomal-backup systems similar to that in mammals have not been observed in the chicken [55]. Moreover the bird ZW and mammalian XY chromosomes have a different ancestral origin and it has been shown that none of the 1000 genes on the chicken Z has an orthologue on the X [56]. Thus, sex-linked genes shown to be toxic when expressed in mammals [47] are not present on the sex chromosomes in birds.

Finally based on our findings we propose that the H2AFX-dependent meiotic silencing mechanism either evolved after the split between mammals and birds, or that it was present in the bird/mammal ancestor and was subsequently lost in the avian lineage. A detailed analysis of meiotic silencing in other organisms will now be essential in order to discriminate between these two possibilities.

## Materials and Methods

### Surface spreads from chicken ovaries and testes

Left ovaries from chicken embryos were dissected at different developmental timepoints and stored at −70°C. Ovaries from hatched chickens were obtained as frozen tissue from Harlan, Cambridge, UK. Spread nuclei were prepared following the protocol of Barlow et al.,1997 [57] with some modifications. Briefly ovaries were minced in PBS using scalpel blades. The cell suspension was dispersed on a glass slide and permeabilised by adding PBS containing 0.05% triton X-100 and 9% sucrose for 10 min. The buffer was drained and the cells fixed in 2% formaldehyde, 0.02% SDS for 1 h. After a rinse in H_2_O the slides were left to air-dry and used immediately for immunohistochemistry or stored at −70°C.

### RNA FISH and antibody staining

BAC clones, carrying autosomal or Z linked genes, were obtained from the CHORI BAC PAC Resource Centre, USA. BAC DNA was extracted from minicultures using the PhasePrep BAC DNA kit (Sigma) and about 2 µg of DNA was labelled with digoxigenin, using a DIG-Nick translation kit (Roche). The DNA probes were purified and used for the RNA FISH experiments as described in Mahadevaiah et al., 2009 [58]. The RNA FISH signal was detected by using anti-DIG-FITC (Chemicon, 1∶10). List of probes: Z1 = BAC CH261-30P24 (RIT2), Z2 = BAC CH261-188G9 (CAPSL, IL7R, LMBRD2), Z3 = BAC CH261-177N9 (DEPDC1B, ELOVL7, ERCC8, NDUFAF2), Z4 = BAC CH261-63L19 (PGM5, MAP1b), Z5 = BAC CH261-125H16 (DMRT1), Z6 = BAC CH261-133M4 (BNC2), Z7 = BAC CH261-135O5 (GNAQ), Z8 = BAC CH261-117H7 (NTRK2), Z9 = BAC CH261-15G5 (HINT1, CDC42SE2, LYRM7, FINX69), on chromosome Z; A1 = BAC CH261-29N14 (SCML2, RAI2), A2 = BAC CH261-88N12 (TAF3, B3VE14, ITIH5), on chromosome 1; A3 = BAC CH261-98L9 (PUM1, SDC3, FAM77c), on chromosome 23.

In order to identify and stage the germ cells, RNA FISH processed slides were rinsed in PBS, blocked for 30 min at room temperature (RT) in PBS, 0.1% Tween-20 (PBST), 0.3% BSA and incubated overnight at 4°C with meiosis specific antibodies: mouse γH2AFX (Upstate, 1∶300) or rabbit SYCP3 (Abcam, 1∶300). The detection was carried out using immunofluorescence as described below. The slides were finally mounted in Vectashield with DAPI (Vector). After imaging, some RNA FISH slides were also stained for RPA-32 using a rabbit polyclonal antibody (Abcam, 1∶300) and images were re-captured.

### DNA FISH and chromosome painting

DNA FISH and chromosome painting were carried out on slides processed for RNA FISH and/or immunofluorescence. DIG-labelled Z and Biotin-labelled W chromosome paints were kindly provided by Drs. D.K. Griffin and M. Völker (University of Kent, Canterbury) [59]. DNA FISH probes were generated from the BACs screened by RNA FISH. 2 µg of BAC DNA was labelled either with the DIG-Nick translation kit, or the BioNick DNA labelling system (Roche), following the manufacturer's instructions. The probes were purified and hybridised to the slides as described in Mahadevaiah et al., 2009 [58]. The DIG signal was detected using anti-DIG-FITC (Chemicon, 1∶10). The biotin signal was detected using avidin AlexaFluor 555 conjugate (Invitrogen, 1∶100), followed by an amplification step with Biotin anti-avidin (Vector, 1∶200) and a final incubation with the avidin AlexaFluor 555 conjugate (Invitrogen, 1∶100).

### Antibodies and immunohistochemistry

Spread slides were incubated for 30 min at RT in blocking buffer (PBST, 0.3% BSA). The slides were then incubated overnight at 37°C with different combinations of primary antibodies diluted in blocking buffer. Following three 5 min washes in PBST at RT, the antibody signal was revealed with Alexa fluorophore conjugated secondary antibodies (Invitrogen 1∶400) in PBST for 1 h at 37°C. After three 5 min washes at RT in PBST, the slides were finally mounted in vectashield +/− DAPI. Fluorescence images were captured on an Olympus IX70 inverted microscope using a Deltavision cooled CCD imaging system (Photometrics CH350L liquid cooled CCD camera; Softworx image acquisition software) (Applied Precision).

Primary antibodies: rabbit SYCP3 (Abcam, 1∶300), rabbit RPA-32 (Abcam, 1∶300), mouse Rad51 (Abcam 1∶200), mouse MLH1 (BD 1∶200), rabbit H3K9me3 (Upstate, 1∶200), rat CBX1 (1∶300, gift from P. Singh, Research Centre Borstel, Borstel, Germany), rabbit TopBP1 (1∶300, gift from J. Chen, University of Texas MD Anderson Cancer Centre, Houston, USA), mouse γH2AFX (Upstate, 1∶200), mouse BrdU (1∶300, Chemicon). Secondary antibodies: Alexa fluorescent anti-mouse, anti-rabbit, and anti-rat 555, anti-rabbit 405, anti-rabbit 488 (Invitrogen).

## Supporting Information

Figure S1 RPA localisation in chicken oocytes. Oocytes stained for RPA (red) and SYCP1 (green). (A) Zygotene, lots of RPA foci, some of which on synaptic chromosomes (indicated with arrows). (B–D) Pachytene; RPA foci are still abundant on all synaptic axes at early pachytene (B), but rapidly decrease by late pachytene (C–D). (C) Late pachytene oocyte RPA negative; (D) Late pachytene oocyte containing a chain of RPA foci SYCP1 negative. Scale bar = 10 µm.
(TIF)

Figure S2 Rad51 localisation in RPA positive late pachytene oocytes. Oocyte stained for Rad51 (red), RPA (green) and SYCP3 (blue). Rad51 foci colocalise with RPA on the unsynapsed chromosome. Scale bar = 10 µm.
(TIF)

Figure S3 TopBP1 localisation in chicken oocytes. Oocytes stained for TopBP1 (red) and SYCP3 (green). (A) Zygotene, TopBP1 foci are on unsynapsed and synapsed chromosomes. (B) Early pachytene; TopBP1 foci are still on the synaptic axes. (C–F) Most late pachytene (C) and early diplotene (E) oocytes are devoided of TopBP1, but a minority of pachytene (D) and diplotene (F) oocytes contain a chain of RPA foci on the asynapsed Z chhromosome. Z and W were identified by hybridisation to Z and W chromosome paints (data not shown). Scale bar = 10 µm.
(TIF)

Table S1 RNA–FISH on oocytes from 1 dph ovaries using probes Z1, Z2, Z3, Z6, Z9. After RNA-FISH the slides were stained for SYCP3 and RPA to identify the germ cells and to distinguish the synaptic cells (RPA^−ve^) from the cells with persistent DSBs (RPA^+ve^). The quantification was done on 50–70 cells identified as RPA^−ve^ and 10–11 cells identified as RPA^+ve^. The percentage of cells found to be positive for the FISH signal is indicated.
(DOC)

## References

[1] Zickler D, Kleckner N (1999). Meiotic chromosomes: integrating structure and function.. Annual review of genetics.

[2] Page J, de la Fuente R, Gomez R, Calvente A, Viera A (2006). Sex chromosomes, synapsis, and cohesins: a complex affair.. Chromosoma.

[3] de la Fuente R, Parra MT, Viera A, Calvente A, Gomez R (2007). Meiotic pairing and segregation of achiasmate sex chromosomes in eutherian mammals: the role of SYCP3 protein.. PLoS Genet.

[4] Stitou S, Jimenez R, Diaz de La Guardia R, Burgos M (2000). Sex-chromosome pairing through heterochromatin in the African rodent Lemniscomys barbarus (Rodentia, Muridae). A synaptonemal complex study.. Chromosome research: an international journal on the molecular, supramolecular and evolutionary aspects of chromosome biology.

[5] Burgoyne PS (1982). Genetic homology and crossing over in the X and Y chromosomes of Mammals.. Human genetics.

[6] McKee BD, Handel MA (1993). Sex chromosomes, recombination, and chromatin conformation.. Chromosoma.

[7] Turner JM (2007). Meiotic sex chromosome inactivation.. Development.

[8] Yan W, McCarrey JR (2009). Sex chromosome inactivation in the male.. Epigenetics: official journal of the DNA Methylation Society.

[9] Baarends WM, Wassenaar E, van der Laan R, Hoogerbrugge J, Sleddens-Linkels E (2005). Silencing of unpaired chromatin and histone H2A ubiquitination in mammalian meiosis.. Molecular and cellular biology.

[10] Turner JM, Mahadevaiah SK, Ellis PJ, Mitchell MJ, Burgoyne PS (2006). Pachytene asynapsis drives meiotic sex chromosome inactivation and leads to substantial postmeiotic repression in spermatids.. Developmental cell.

[11] Turner JM, Mahadevaiah SK, Fernandez-Capetillo O, Nussenzweig A, Xu X (2005). Silencing of unsynapsed meiotic chromosomes in the mouse.. Nat Genet.

[12] Schimenti J (2005). Synapsis or silence.. Nature genetics.

[13] Inagaki A, Schoenmakers S, Baarends WM (2010). DNA double strand break repair, chromosome synapsis and transcriptional silencing in meiosis.. Epigenetics: official journal of the DNA Methylation Society.

[14] Shiu PK, Raju NB, Zickler D, Metzenberg RL (2001). Meiotic silencing by unpaired DNA.. Cell.

[15] Bean CJ, Schaner CE, Kelly WG (2004). Meiotic pairing and imprinted X chromatin assembly in Caenorhabditis elegans.. Nature genetics.

[16] Garcia-Cruz R, Roig I, Robles P, Scherthan H, Garcia Caldes M (2009). ATR, BRCA1 and gammaH2AX localize to unsynapsed chromosomes at the pachytene stage in human oocytes.. Reproductive biomedicine online.

[17] Hense W, Baines JF, Parsch J (2007). X chromosome inactivation during Drosophila spermatogenesis.. PLoS Biol.

[18] Vibranovski MD, Lopes HF, Karr TL, Long M (2009). Stage-specific expression profiling of Drosophila spermatogenesis suggests that meiotic sex chromosome inactivation drives genomic relocation of testis-expressed genes.. PLoS Genet.

[19] Mikhaylova LM, Nurminsky DI (2011). Lack of global meiotic sex chromosome inactivation, and paucity of tissue-specific gene expression on the Drosophila X chromosome.. BMC biology.

[20] Turner JM, Aprelikova O, Xu X, Wang R, Kim S (2004). BRCA1, histone H2AX phosphorylation, and male meiotic sex chromosome inactivation.. Current biology: CB.

[21] Ichijima Y, Ichijima M, Lou Z, Nussenzweig A, Camerini-Otero RD (2011). MDC1 directs chromosome-wide silencing of the sex chromosomes in male germ cells.. Genes & development.

[22] Fernandez-Capetillo O, Mahadevaiah SK, Celeste A, Romanienko PJ, Camerini-Otero RD (2003). H2AX is required for chromatin remodeling and inactivation of sex chromosomes in male mouse meiosis.. Dev Cell.

[23] Kelly WG, Aramayo R (2007). Meiotic silencing and the epigenetics of sex.. Chromosome research: an international journal on the molecular, supramolecular and evolutionary aspects of chromosome biology.

[24] Maine EM (2010). Meiotic silencing in Caenorhabditis elegans.. International review of cell and molecular biology.

[25] Namekawa SH, VandeBerg JL, McCarrey JR, Lee JT (2007). Sex chromosome silencing in the marsupial male germ line.. Proceedings of the National Academy of Sciences of the United States of America.

[26] Solari AJ (1992). Equalization of Z and W axes in chicken and quail oocytes.. Cytogenetics and cell genetics.

[27] Solari AJ, Pigozzi MI (1993). Recombination nodules and axial equalization in the ZW pairs of the Peking duck and the guinea fowl.. Cytogenetics and cell genetics.

[28] Schoenmakers S, Wassenaar E, Hoogerbrugge JW, Laven JS, Grootegoed JA (2009). Female meiotic sex chromosome inactivation in chicken.. PLoS Genet.

[29] Lammers JH, Offenberg HH, van Aalderen M, Vink AC, Dietrich AJ (1994). The gene encoding a major component of the lateral elements of synaptonemal complexes of the rat is related to X-linked lymphocyte-regulated genes.. Mol Cell Biol.

[30] Moens PB, Kolas NK, Tarsounas M, Marcon E, Cohen PE (2002). The time course and chromosomal localization of recombination-related proteins at meiosis in the mouse are compatible with models that can resolve the early DNA-DNA interactions without reciprocal recombination.. J Cell Sci.

[31] Plug AW, Peters AH, Keegan KS, Hoekstra MF, de Boer P (1998). Changes in protein composition of meiotic nodules during mammalian meiosis.. J Cell Sci.

[32] Moens PB, Marcon E, Shore JS, Kochakpour N, Spyropoulos B (2007). Initiation and resolution of interhomolog connections: crossover and non-crossover sites along mouse synaptonemal complexes.. J Cell Sci.

[33] Ashley T (2000). An integration of old and new perspectives of mammalian meiotic sterility.. Results and problems in cell differentiation.

[34] Sun J, Oma Y, Harata M, Kono K, Shima H (2010). ATM modulates the loading of recombination proteins onto a chromosomal translocation breakpoint hotspot.. PLoS ONE.

[35] Anderson LK, Reeves A, Webb LM, Ashley T (1999). Distribution of crossing over on mouse synaptonemal complexes using immunofluorescent localization of MLH1 protein.. Genetics.

[36] Pigozzi MI, Solari AJ (1999). Equal frequencies of recombination nodules in both sexes of the pigeon suggest a basic difference with eutherian mammals.. Genome/National Research Council Canada = Genome/Conseil national de recherches Canada.

[37] Mahadevaiah SK, Turner JM, Baudat F, Rogakou EP, de Boer P (2001). Recombinational DNA double-strand breaks in mice precede synapsis.. Nat Genet.

[38] Perera D, Perez-Hidalgo L, Moens PB, Reini K, Lakin N (2004). TopBP1 and ATR colocalization at meiotic chromosomes: role of TopBP1/Cut5 in the meiotic recombination checkpoint.. Molecular biology of the cell.

[39] Motzkus D, Singh PB, Hoyer-Fender S (1999). M31, a murine homolog of Drosophila HP1, is concentrated in the XY body during spermatogenesis.. Cytogenetics and cell genetics.

[40] Martens JH, O'Sullivan RJ, Braunschweig U, Opravil S, Radolf M (2005). The profile of repeat-associated histone lysine methylation states in the mouse epigenome.. The EMBO journal.

[41] Peters AH, Kubicek S, Mechtler K, O'Sullivan RJ, Derijck AA (2003). Partitioning and plasticity of repressive histone methylation states in mammalian chromatin.. Molecular cell.

[42] Burgoyne PS, Mahadevaiah SK, Turner JM (2009). The consequences of asynapsis for mammalian meiosis.. Nature reviews Genetics.

[43] Rodriguez TA, Burgoyne PS (2000). Evidence that sex chromosome asynapsis, rather than excess Y gene dosage, is responsible for the meiotic impairment of XYY mice.. Cytogenetics and cell genetics.

[44] Setterfield LA, Mahadevaiah S, Mittwoch U (1988). Chromosome pairing and germ cell loss in male and female mice carrying a reciprocal translocation.. Journal of reproduction and fertility.

[45] Roeder GS, Bailis JM (2000). The pachytene checkpoint.. Trends in genetics: TIG.

[46] Li XC, Schimenti JC (2007). Mouse pachytene checkpoint 2 (trip13) is required for completing meiotic recombination but not synapsis.. PLoS Genet.

[47] Royo H, Polikiewicz G, Mahadevaiah SK, Prosser H, Mitchell M (2010). Evidence that meiotic sex chromosome inactivation is essential for male fertility.. Current biology: CB.

[48] Deckbar D, Birraux J, Krempler A, Tchouandong L, Beucher A (2007). Chromosome breakage after G2 checkpoint release.. The Journal of cell biology.

[49] Hunt PA, Hassold TJ (2008). Human female meiosis: what makes a good egg go bad?. Trends in genetics: TIG.

[50] Vernet N, Mahadevaiah SK, Ojarikre OA, Longepied G, Prosser HM (2011). The Y-encoded gene zfy2 acts to remove cells with unpaired chromosomes at the first meiotic metaphase in male mice.. Current biology: CB.

[51] Mahadevaiah SK, Royo H, VandeBerg JL, McCarrey JR, Mackay S (2009). Key features of the X inactivation process are conserved between marsupials and eutherians.. Current biology: CB.

[52] Mueller JL, Mahadevaiah SK, Park PJ, Warburton PE, Page DC (2008). The mouse X chromosome is enriched for multicopy testis genes showing postmeiotic expression.. Nature genetics.

[53] Namekawa SH, Park PJ, Zhang LF, Shima JE, McCarrey JR (2006). Postmeiotic sex chromatin in the male germline of mice.. Current biology: CB.

[54] Wang PJ (2004). X chromosomes, retrogenes and their role in male reproduction.. Trends in endocrinology and metabolism: TEM.

[55] ICGS Consortium (2004). Sequence and comparative analysis of the chicken genome provide unique perspectives on vertebrate evolution.. Nature.

[56] Bellott DW, Skaletsky H, Pyntikova T, Mardis ER, Graves T (2010). Convergent evolution of chicken Z and human X chromosomes by expansion and gene acquisition.. Nature.

[57] Barlow AL, Benson FE, West SC, Hulten MA (1997). Distribution of the Rad51 recombinase in human and mouse spermatocytes.. EMBO J.

[58] Mahadevaiah SK, Costa Y, Turner JM (2009). Using RNA FISH to study gene expression during mammalian meiosis.. Methods in molecular biology.

[59] Shetty S, Griffin DK, Graves JA (1999). Comparative painting reveals strong chromosome homology over 80 million years of bird evolution.. Chromosome research: an international journal on the molecular, supramolecular and evolutionary aspects of chromosome biology.

