# Supplementary figures and images for: Error-Prone ZW Pairing and No Evidence for Meiotic Sex Chromosome Inactivation in the Chicken Germ Line
Source: PLoS Genet. 2012 Mar 8;8(3):e1002560. doi: 10.1371/journal.pgen.1002560 (PMC3297585; doi:10.1371/journal.pgen.1002560)

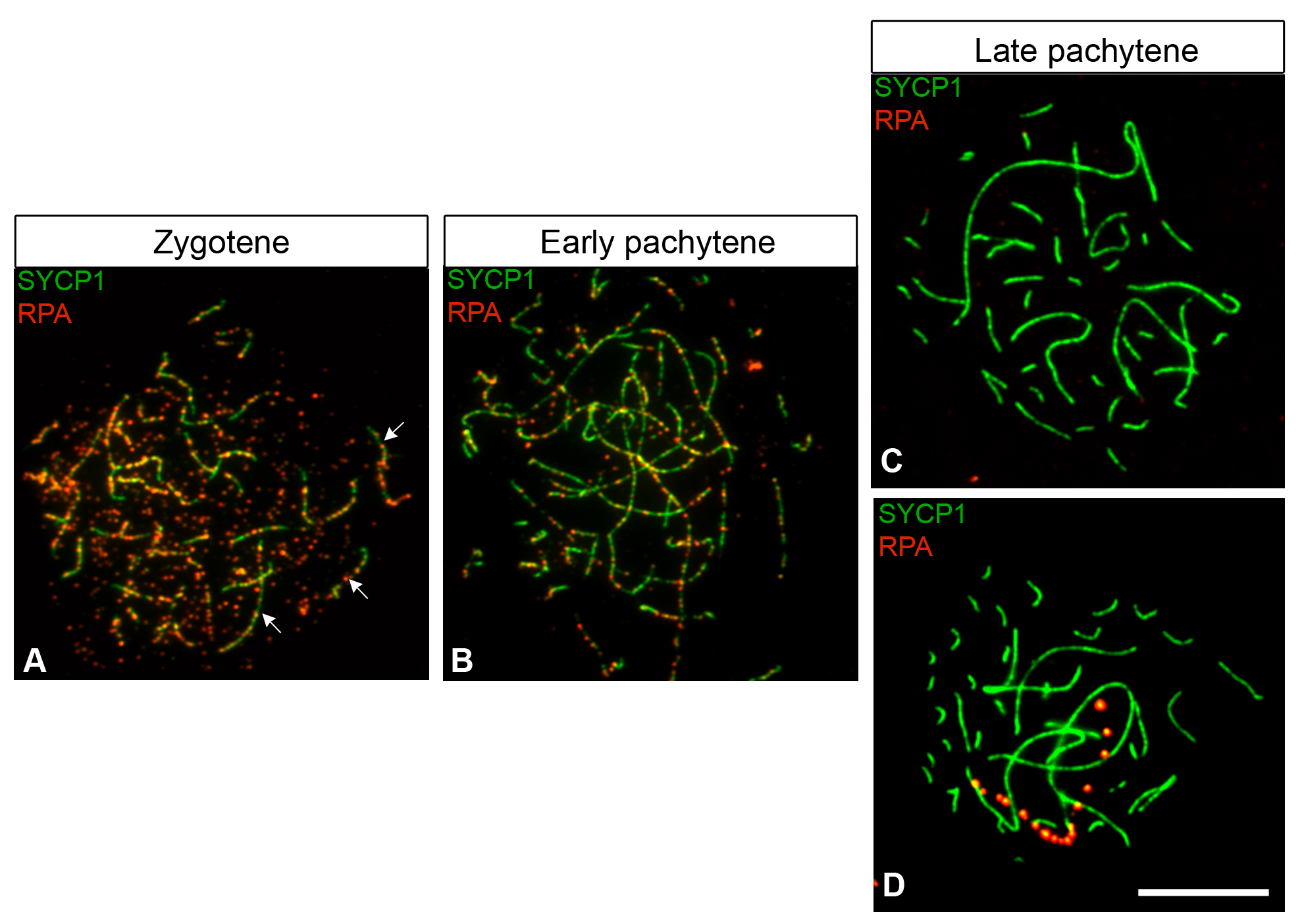

Supplement: Figure S1 — RPA localisation in chicken oocytes. Oocytes stained for RPA (red) and SYCP1 (green). (A) Zygotene, lots of RPA foci, some of which on synaptic chromosomes (indicated with arrows). (B–D) Pachytene; RPA foci are still abundant on all synaptic axes at early pachytene (B), but rapidly decrease by late pachytene (C–D). (C) Late pachytene oocyte RPA negative; (D) Late pachytene oocyte containing a chain of RPA foci SYCP1 negative. Scale bar = 10 µm. (TIF) [file pgen.1002560.s001.tif]

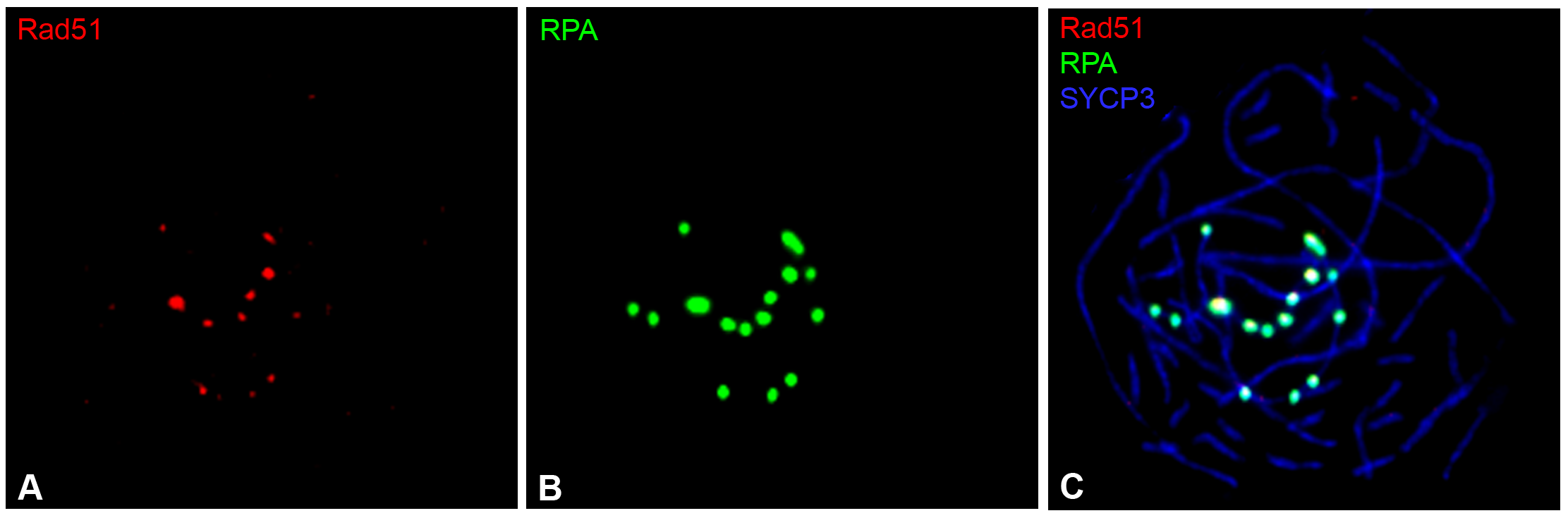

Supplement: Figure S2 — Rad51 localisation in RPA positive late pachytene oocytes. Oocyte stained for Rad51 (red), RPA (green) and SYCP3 (blue). Rad51 foci colocalise with RPA on the unsynapsed chromosome. Scale bar = 10 µm. (TIF) [file pgen.1002560.s002.tif]

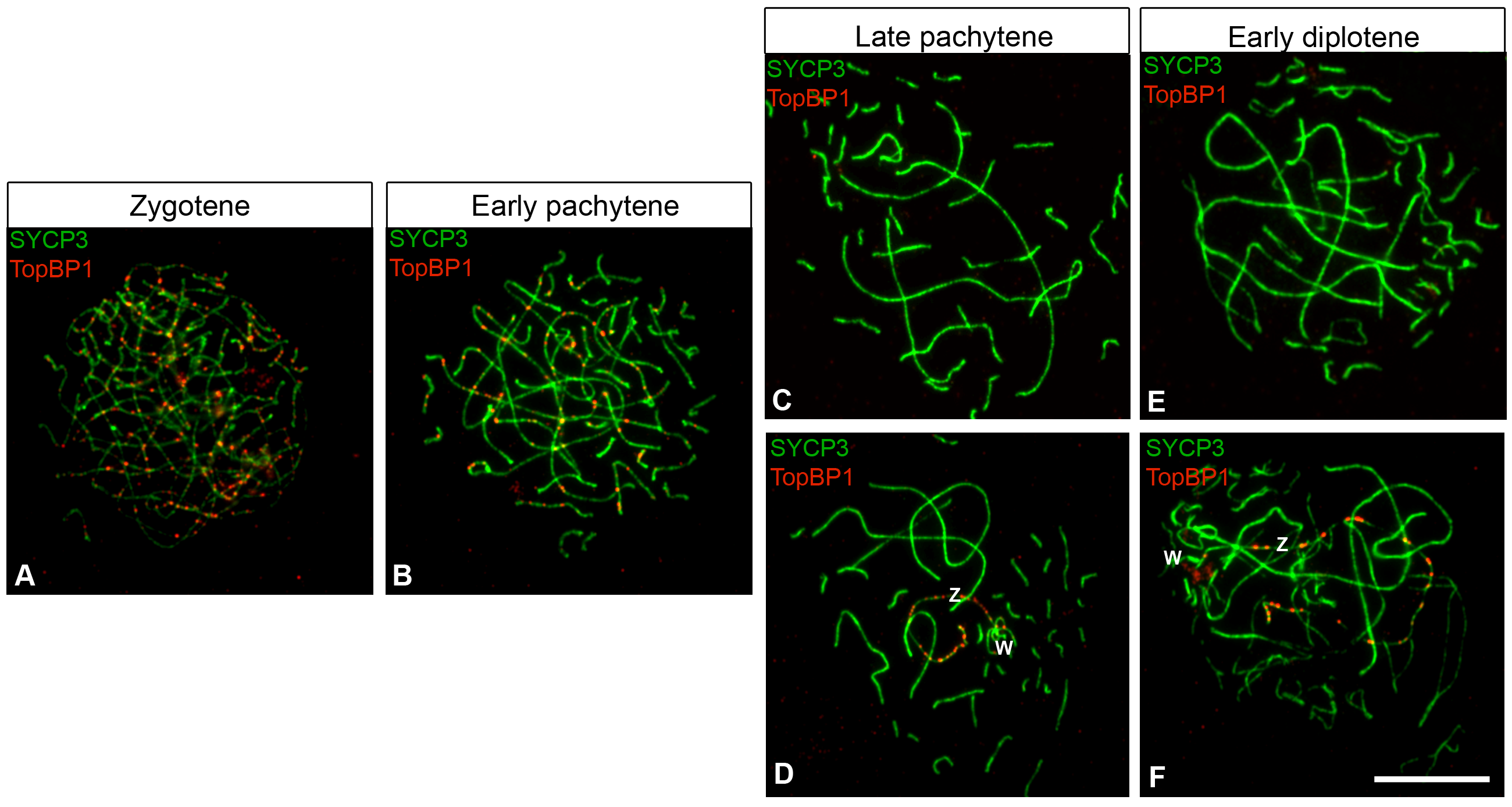

Supplement: Figure S3 — TopBP1 localisation in chicken oocytes. Oocytes stained for TopBP1 (red) and SYCP3 (green). (A) Zygotene, TopBP1 foci are on unsynapsed and synapsed chromosomes. (B) Early pachytene; TopBP1 foci are still on the synaptic axes. (C–F) Most late pachytene (C) and early diplotene (E) oocytes are devoided of TopBP1, but a minority of pachytene (D) and diplotene (F) oocytes contain a chain of RPA foci on the asynapsed Z chhromosome. Z and W were identified by hybridisation to Z and W chromosome paints (data not shown). Scale bar = 10 µm. (TIF) [file pgen.1002560.s003.tif]
