# Supplementary material for: Error-Prone ZW Pairing and No Evidence for Meiotic Sex Chromosome Inactivation in the Chicken Germ Line
Source: PLoS Genet. 2012 Mar 8;8(3):e1002560. doi: 10.1371/journal.pgen.1002560 (PMC3297585; doi:10.1371/journal.pgen.1002560)
Supplement: Table S1 — RNA–FISH on oocytes from 1 dph ovaries using probes Z1, Z2, Z3, Z6, Z9. After RNA-FISH the slides were stained for SYCP3 and RPA to identify the germ cells and to distinguish the synaptic cells (RPA−ve) from the cells with persistent DSBs (RPA+ve). The quantification was done on 50–70 cells identified as RPA−ve and 10–11 cells identified as RPA+ve. The percentage of cells found to be positive for the FISH signal is indicated. (DOC) [file pgen.1002560.s004.doc]

| **Z BAC probes** | **% of RPA^-ve^ cells FISH+** | **% of RPA^+ve^ cells FISH+** |
| --- | --- | --- |
| Z1 | 34 | 30 |
| Z2 | 27 | 30 |
| Z3 | 12 | 10 |
| Z6 | 25 | 18 |
| Z9 | 24 | 30 |
